# Robotic-assisted plate fixation of the anterior acetabulum - clinical description of a new technique

**DOI:** 10.1186/s13018-024-04731-x

**Published:** 2024-04-22

**Authors:** Koroush Kabir, Friedrich-Carl von Rundstedt, Jonas Roos, Martin Gathen

**Affiliations:** 1 Centre of Trauma and Orthopaedic Surgery, Helios University Clinic Wuppertal, Wuppertal, Germany; 2 Department of Urology, Helios University Hospital Wuppertal, University of Witten/Herdecke, Wuppertal, Germany; 3 Department of Orthopedics and Trauma Surgery, University Hospital of Bonn, Venusberg-Campus 1, Bonn, 53127 Germany

**Keywords:** Pelvic ring, Acetabulum, Plate fixation, Minimally invasive approach, Robotic-assisted surgery

## Abstract

**Introduction:**

We present a detailed procedure for the robotic-assisted plate osteosynthesis of an anterior acetabular fracture. The purpose of this work was to describe a robotic-assisted minimally invasive technique as a possible method for reducing complications, pain, and hospitalization. Another goal was to present technical recommendations and to assess potential pitfalls and problems of the new surgical approach.

**Methods:**

Surgery was performed in an interdisciplinary setting by an experienced orthopedic surgeon and a urologist. The DaVinci System with standard instruments was used. Reduction was achieved through indirect traction of a pin that was introduced into the femoral neck and direct manipulation via the plate. The plate position and fixation were achieved through 7 additional minimally invasive incisions.

**Results:**

The technique has multiple advantages, such as no detachment of the rectus abdominal muscle, a small skin incision, and minimal blood loss. Furthermore, this approach might lower the incidence of hernia formation, infection, and postoperative pain.

**Discussion:**

We see the presented technique as a demanding yet progressive and innovative surgical method for treating acetabular fractures with indications for anterior plate fixation.

**Trial registration:**

The study was approved by the local institutional review board (Nr. 248/18).

## Introduction

Acetabular fractures are demanding injuries even when they are treated in specialized centers and by experienced surgeons. Large incisions such as the ilioinguinal approach are still the worldwide standard approach for anterior wall and anterior column fractures [1]. It allows wide access to the acetabulum but can be associated with significant complication rates of up to 31% [2]. The complex anatomy of the pelvis and the required approach can lead to relevant bleeding, infection or posttraumatic arthritis, resulting in a suboptimal recovery or outcome [3]. Recent studies have described minimally invasive and innovative laparoscopic techniques with the goal of reducing common complications by using smaller incisions and sparing the ability to detach muscles [4, 5]. This technique may also reduce the risk of iatrogenic neurovascular injuries due to excellent visualization [6]. The next logical step to optimize and standardize the procedure and allow better handling is the use of a robotic-assisted surgical system. Modern robotic systems have gained widespread acceptance for multiple surgical procedures and offer 3D vision systems, enhanced dexterity and safe control for surgeons.

We present an innovative robotic-assisted surgical approach for minimally invasive internal fixation of the anterior acetabulum and share our experience, tips and tricks.

## Surgical technique

### Patient

**Fig. 1:**
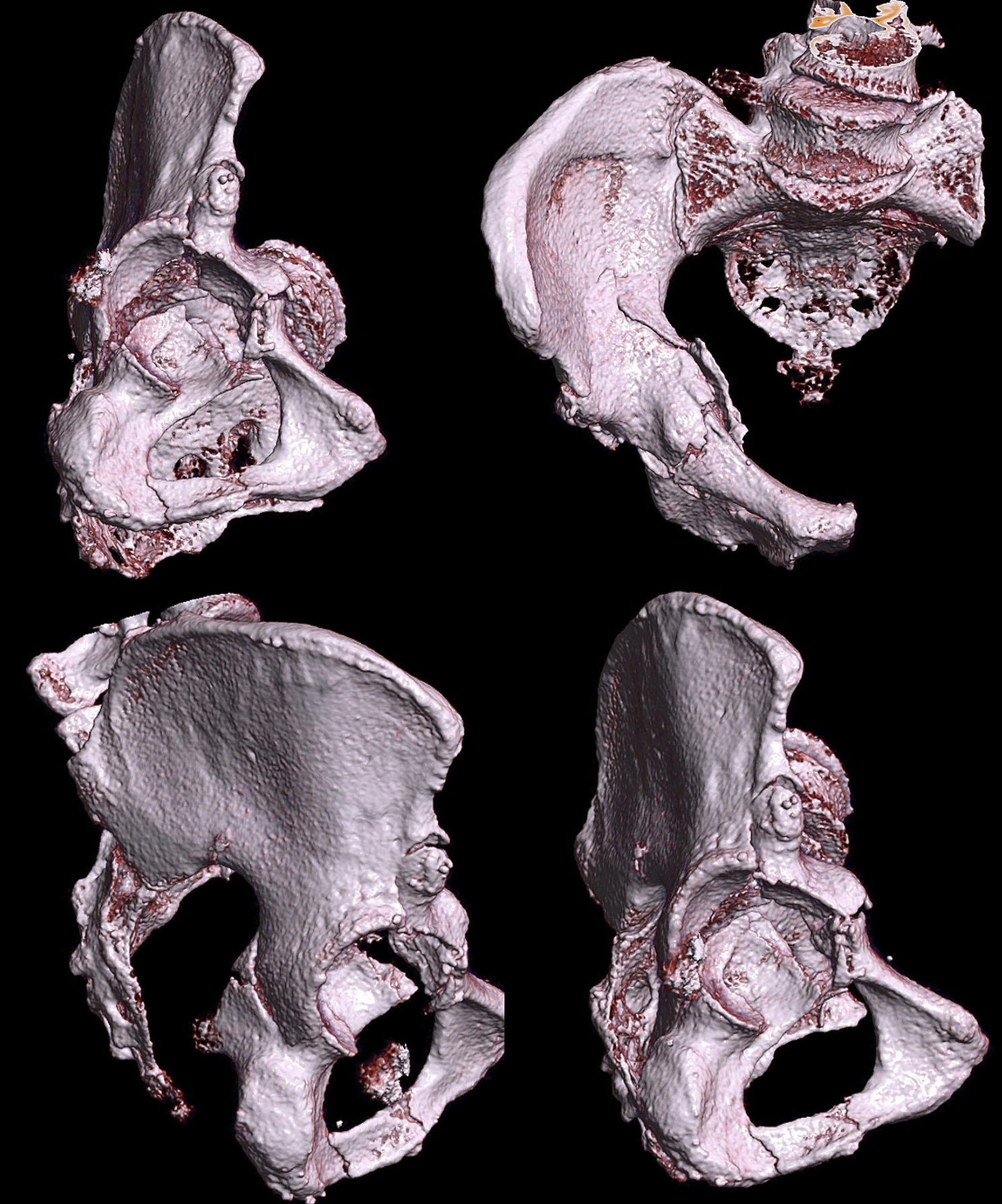
The illustration displays the 3D reconstruction of the fracture morphology based on preoperative CT imaging. The image revealed an anterior column and hemitransverse fracture of the right acetabulum

The patient was an 87-year-old male who suffered an anterior column and posterior hemitransverse fracture of the right acetabulum through a simple fall (Fig. 1). Informed consent regarding the innovative nature of the technique was obtained from the patient. The patient was informed about alternative therapies and received an individual information sheet. The surgical procedure was planned and performed in an interdisciplinary setting by an experienced orthopedic surgeon and a urologist. The study was approved by the Medical Ethics Committee of our institution (University Ethics Committee No. 248/18).

### Approach

**Fig. 2:**
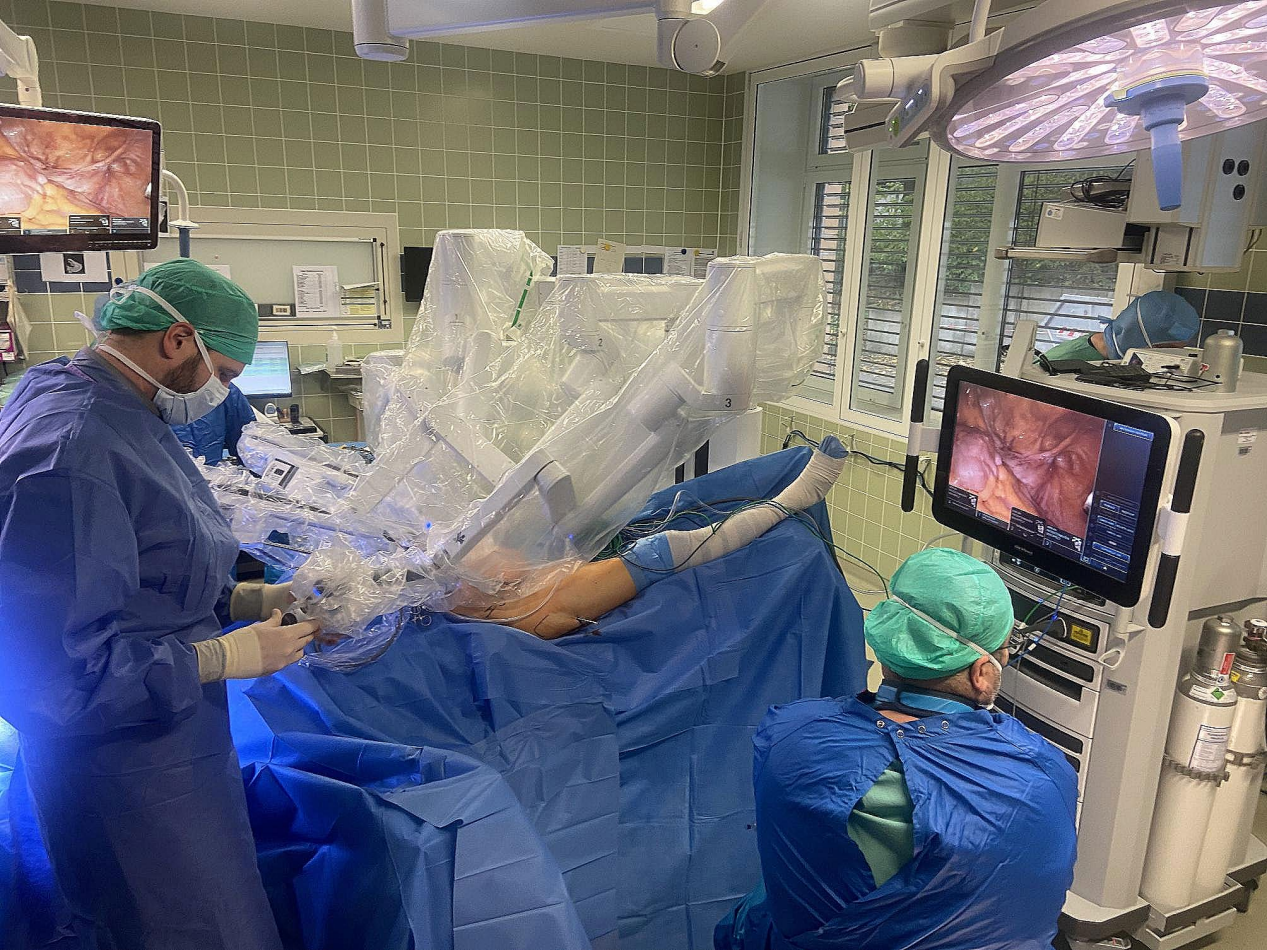
The figure shows the placement of the DaVinci robot in the operating theater during the preparation of the surgical site by the urological surgeon

The patient is placed in a supine position on a carbon table that allows easy imaging by image intensifier fluoroscopy. Before surgery, fluoroscopic AP, inlet, outlet and Judet oblique views X-rays were obtained to ensure adequate visualization of the fracture site. A supraumbilical longitudinal incision was made to insert the 8 mm camera trocar. This was followed by visualization of the fascia and puncture of the abdominal cavity using a Veress needle. After elevating the abdominal wall and verifying its correct positioning, CO2 was insufflated to a pressure of 12 cm H2O. Subsequently, a second camera trocar was introduced cranially to the navel. Working trocars for the manipulator were positioned approximately 9 cm lateral to the midline just below the navel. The lateral screws, for example, could also be inserted via this access during the operation, so that no further accesses were necessary. The 4th trocar for the manipulator was placed level with the camera trocar, 9 cm from the other trocar on the left side. Lateral and medial to the right trocar, another two trocars (12 and 5 mm) were placed slightly more cranially. The position was then adjusted to a head-down tilt, and the DaVinci system was used for docking. Figure 2 shows an overview of the placement of the Da Vinci robot in the operating theater.

### Surgical technique

The operation began with the placement of a Steinmann screw (DePuy Synthes, Raynham, MA, USA) in the right femoral neck. Under fluoroscopic control, height localization was achieved. A surgical incision was made on the lateral thigh, below the trochanter, and a Steinmann screw was placed in the femoral neck.

Then, the dorsolateral parietal peritoneum was incised, allowing for the predominant blunt presentation of the Cavum Retzi and the right fossa. The bladder was retracted medially, and the peritoneal sac was retracted cranially. Dissection of the external iliac artery commenced at the internal inguinal ring and continued to the origin of the internal iliac artery. Both vessels were looped. Two dorsally running vessels (smaller vessels) were ligated, enabling the external iliac vein and artery to be mobilized laterally and cranially for subsequent plate placement. A significant amount of fatty tissue was found in this area. The lymphatic channels were coagulated, and the medial lymphatic bundle was clipped distally. The lymphatic tissue was then rolled off the vein, revealing the obturator fossa. A hematoma was observed here. Further deep dissection was performed to expose the femoral head. The hematoma was flushed and debrided.

A skin incision of approximately 3 cm in length was made horizontally above the symphysis. Subcutaneous dissection was carried out with successive hemostasis. Dissection continued until the fascia was reached, followed by the placement of a trocar. Meticulous dissection along the superior pubic ramus and the infrapectineal line was performed dorsally up to the sacroiliac joint. The ‘corona mortis’ was identified and ligated using clips. The obturator vessels were identified and preserved. The quadrilaminar surface, which was notably protruded and internally fractured, was cleaned of hematoma. The area was then flushed for a better overview.

### Reduction

**Fig. 3:**
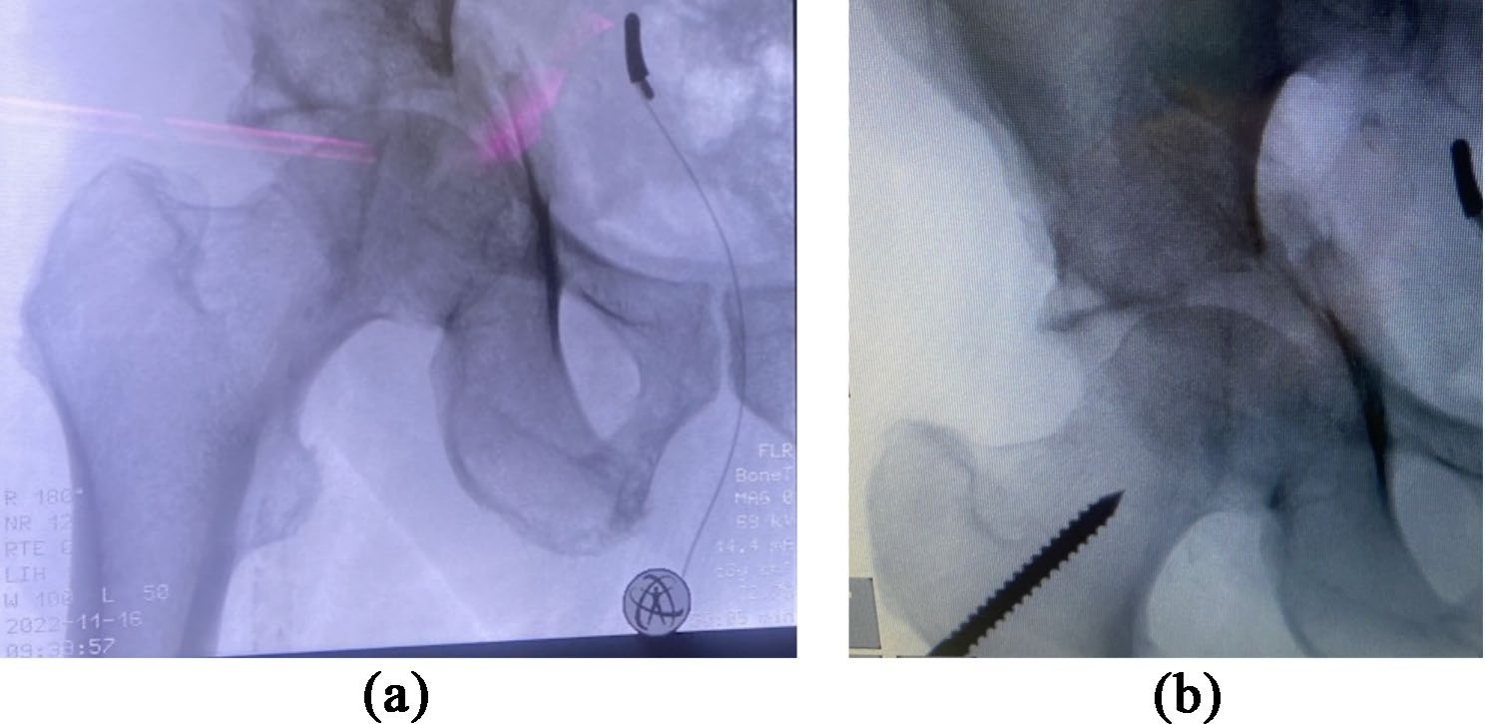
The left image (**3a**) displays the intraoperative X-ray image without repositioning. The right image (**3b**) shows the initial repositioning under traction applied by the Steinmann screw

While traction was applied to the right leg using the Steinmann screw, the fragment complex was repositioned laterally with concurrent pressure applied using a ball-tipped probe. Ultimately, anatomical repositioning was achieved, and the device was secured using a 1.8 mm K-wire. Another K-wire was placed directly under visualization below the external iliac vein and artery to keep the vessels aside. Figure 3a-b shows the intraoperative X-rays before and after reduction.

### Fixation

**Fig. 4:**
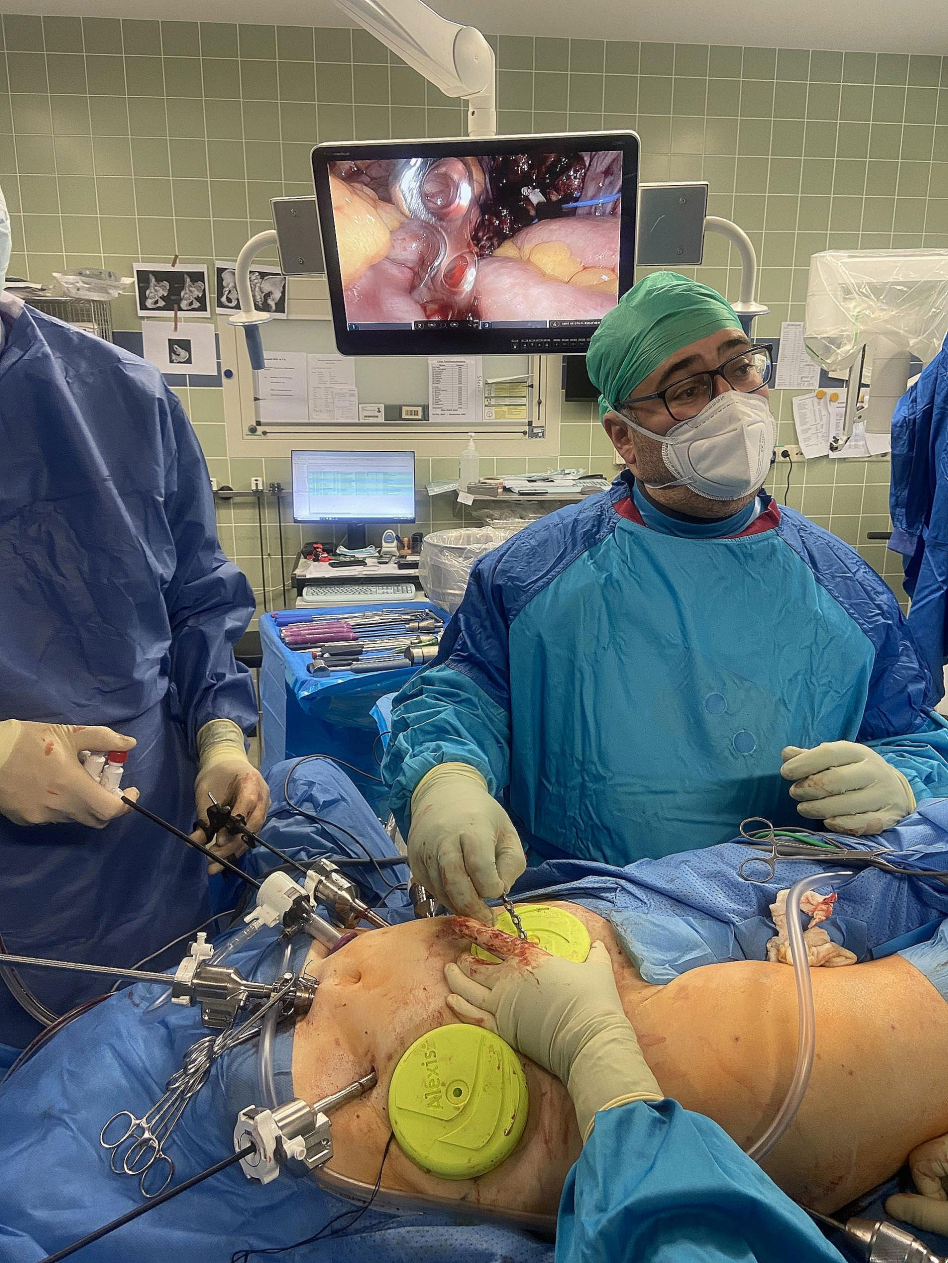
Placement of the plate in the operating theater via the suprapubic approach

Through suprapubic access, a suprapectineal plate (Pro pelvis and Acetabular System, Stryker, MI, USA) is placed endoscopically below the vessels. Figure 4 illustrates the intraoperative placement of the plate via the suprapubic approach. Currently, under endoscopic guidance, placement starts with a ventral screw, which is occupied with appropriate 3.5 mm cortex screws. After the insertion of the first screw, which was not fully tightened, the plate position could be finely adjusted. Subsequently, the first screw was fully tightened, and the plate was further secured with additional screws in the usual manner (3 near the symphysis and 3 in the ilium).

All screws had good anchorage in the bone at the final stage. Fluoroscopic control (A.P., lateral, iliac, and obturaor) revealed good implant positioning and, considering fragmentation, satisfactory reduction. There was no intra-articular friction when moving the joint. The operation took 312 min and the blood loss was approx. 250 ml.

### Aftercare

**Fig. 5:**
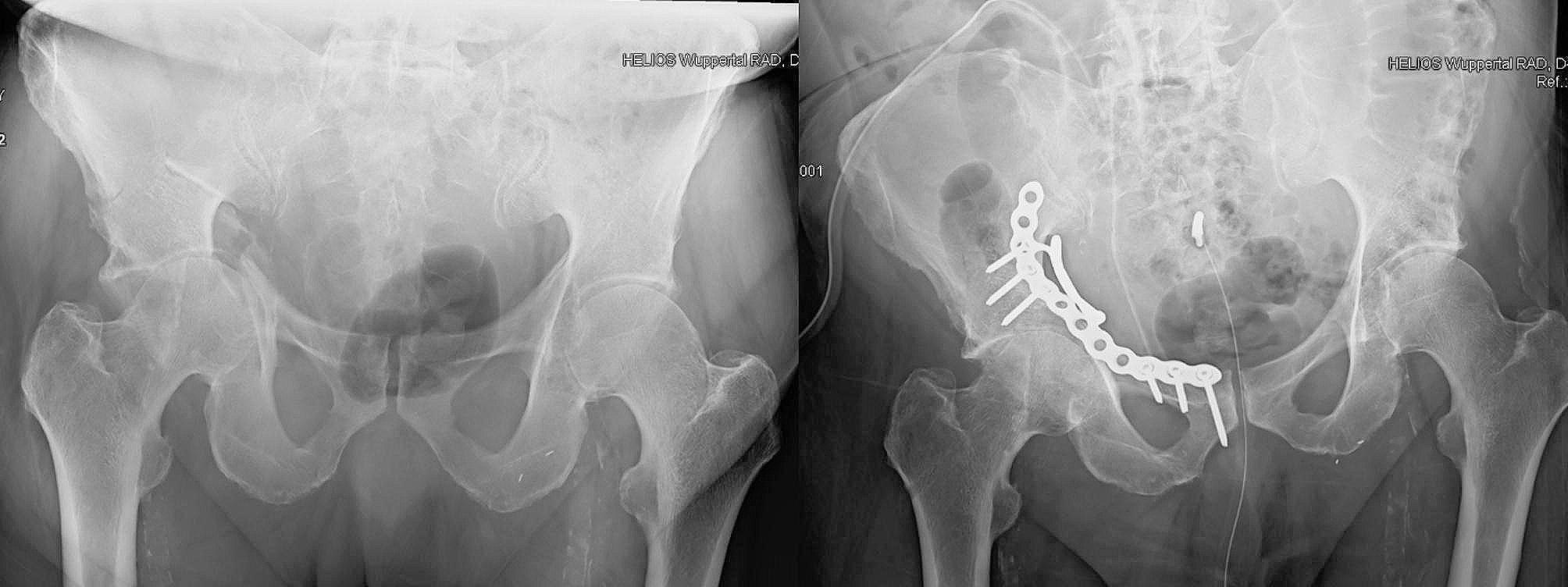
Preoperative X-ray image showing the fracture on the left and postoperative X-ray image showing the correctly positioned implant on the right

No weight-bearing was allowed for the affected lower extremity six weeks after surgery. Follow-up examinations and X-ray control were performed after 6 and 12 weeks. Figure 5 shows the postoperative X-ray image of the patient who underwent inserted osteosynthesis. There was no restriction on the mobility of the affected joints, and patients were allowed to sit up straight. We recommended consequent physiotherapy and applied thrombosis prophylaxis with low-molecular heparin until full remobilization. The patient did not suffer any postoperative complications and the healing process was regular.

## Discussion

Robotic-assisted surgery has recently emerged as an alternative minimally invasive surgical option [7]. For the procedure, laparoscopic instruments are connected to a robotic device controlled by a surgeon via a remote console. Robotic-assisted surgery allows 3-dimensional visualization and enhanced handling of the instrumentation and small incisions [7]. The technique first gained widespread recognition and application in radical prostatectomy and showed lower complication rates than did the open retropubic approach [8]. In the last decade, the use of a surgical robot has been described for a variety of other procedures, such as robotic assisted hemi-colectomy or hysterectomy [9, 10].

The use of this technique for fracture treatment has rarely been described. A systematic review concerning robot-assisted fracture fixation in orthopedic trauma surgery from 2021 included a total of 8 studies [11]. Seven studies described percutaneous screw fixation, and one described intramedullary nail fixation. Thus, robotic-assisted plate fixation is a completely new approach. In this study, the technique was used for anterior plate fixation of the acetabulum. The extrapelvic ilioinguinal approach is still acknowledged as the gold standard for open reduction and internal fixation of acetabular fractures involving the anterior wall or column. A less invasive alternative is the intrapelvic or modified Stoppa approach, which is mostly combined with the first window of the ilioinguinal approach [12]. However, large incisions in the pelvic region can be accompanied by infections on the surgical side, postoperative pain or iatrogenic neurovascular damage [2, 13, 14]. Therefore, less invasive techniques, such as the pararectus approach, were described by Keel et al. [12]. The authors use describe a single inzision along the lateral border of the rectus abdominis muscle followed by an extraperitoneal approach to the acetabulum. Furthermore, minimally invasive laparoscopic approaches for accessing the acetabulum and anterior pelvic ring have recently been described [4, 15]. Techniques that are regularly used for the treatment of hernias to address symphyseal disruption or anterior acetabulum fractures are utilized. In an anatomical feasibility study, Kueper et al. prepared the complete anterior column, including the quadrilateral surface, using the DaVinci system [16].

In conclusion, we identified robotic-assisted plate fixation as a promising alternative for bony injuries of the anterior pelvic ring that allows optimal visualization in combination with minimal incisions.

## Data Availability

No datasets were generated or analysed during the current study.
